# The extracellular matrix controls gap junction protein expression and function in postnatal hippocampal neural progenitor cells

**DOI:** 10.1186/1471-2202-10-13

**Published:** 2009-02-24

**Authors:** Sophie Imbeault, Lianne G Gauvin, Hadi D Toeg, Alexandra Pettit, Catherine D Sorbara, Lamiaa Migahed, Rebecca DesRoches, A Sheila Menzies, Kiyomasa Nishii, David L Paul, Alexander M Simon, Steffany AL Bennett

**Affiliations:** 1 Neural Regeneration Laboratory and Ottawa Institute of Systems Biology, Dept. of Biochemistry, Microbiology, and Immunology, University of Ottawa, ON, Canada; 2 Dept. of Neurobiology, Harvard Medical School, Boston, MA, USA; 3 Dept. of Cellular Neurobiology, Graduate School of Medicine, University of Tokyo, Tokyo, Japan; 4 Dept. of Physiology, University of Arizona, Tucson, AZ, USA

## Abstract

**Background:**

Gap junction protein and extracellular matrix signalling systems act in concert to influence developmental specification of neural stem and progenitor cells. It is not known how these two signalling systems interact. Here, we examined the role of ECM components in regulating connexin expression and function in postnatal hippocampal progenitor cells.

**Results:**

We found that Cx26, Cx29, Cx30, Cx37, Cx40, Cx43, Cx45, and Cx47 mRNA and protein but only Cx32 and Cx36 mRNA are detected in distinct neural progenitor cell populations cultured in the absence of exogenous ECM. Multipotential Type 1 cells express Cx26, Cx30, and Cx43 protein. Their Type 2a progeny but not Type 2b and 3 neuronally committed progenitor cells additionally express Cx37, Cx40, and Cx45. Cx29 and Cx47 protein is detected in early oligodendrocyte progenitors and mature oligodendrocytes respectively. Engagement with a laminin substrate markedly increases Cx26 protein expression, decreases Cx40, Cx43, Cx45, and Cx47 protein expression, and alters subcellular localization of Cx30. These changes are associated with decreased neurogenesis. Further, laminin elicits the appearance of Cx32 protein in early oligodendrocyte progenitors and Cx36 protein in immature neurons. These changes impact upon functional connexin-mediated hemichannel activity but not gap junctional intercellular communication.

**Conclusion:**

Together, these findings demonstrate a new role for extracellular matrix-cell interaction, specifically laminin, in the regulation of intrinsic connexin expression and function in postnatal neural progenitor cells.

## Background

Juxtacrine signalling mechanisms, specifically cell-extracellular matrix (ECM) interactions and gap junctional intercellular communication (GJIC), act in concert to influence developmental specification of neural stem and progenitor cells (NPCs). The interaction of laminins with α1β6 integrin receptors segregates proliferating units in the embryonic ventricular zone from migrating units in the overlying cortex [1] while GJIC within these ECM-defined boundaries ensures synchronous cellular activity between cells destined to become functional domains [2-4]. Similarly, the interaction of laminin with receptors containing β1 integrin directs radial migration of proliferating neuroblasts from the embryonic ventricular zone and along the adult rostral migratory pathway [1,5] while the presence of gap junction connexin proteins facilitates migration of neuroblasts along the axial processes of their radial glial guides during cortical lamination [6-8]. Thus, available data suggest that ECM and connexin-mediated signalling systems act synergistically to direct NPC specialization and migration.

Gap junction communication occurs through intercellular channels formed by connexins [9]. Oligomerization of six connexins forms a hemichannel (connexon) when inserted into non-junctional plasma membranes [10]. Connexons allow for the regulated passage of ions and small molecules (≤ 1 kDa) between the cytoplasm and the extracellular space [10]. Alignment and docking of two connexons in adjacent cells creates an intercellular channel enabling direct cell-cell communication and possibly adhesion. Clusters of intercellular channels make up the morphologically defined gap junction [9]. There are 20 different connexin proteins in mouse; 21 in humans [11]. Fourteen (Cx26, Cx29, Cx30, Cx30.2, Cx31.1, Cx31.9, Cx32, Cx36, Cx37, Cx40, Cx43, Cx45, Cx47, Cx57) are detected in embryonic and/or adult central nervous system [12-15] but only select connexin combinations are capable of oligomerization, adhesion, and functional communication.

The impact of ECM components upon connexin expression in NPCs has not been tested. This is an important issue because NPCs are routinely expanded as neurospheres in the absence of exogenous ECM while functional assessment of cell-cell signalling pathways often involves replating on adhesive substrates. Understanding the changes in NPC phenotype in 2D and 3D culture systems is necessary if we are to rigorously test and validate potential strategies involving NPC expansion and specification *in vitro *[16]. It has already been demonstrated, in other cell types, that connexin expression and intercellular communication is strongly influenced by laminin-integrin interactions [17-19]. In this study, we sought to identify the connexins intrinsically expressed by postnatal NPC populations and determine whether exposure to laminin or simple adhesion alters connexin expression and connexin-mediated GJIC and/or hemichannel activity. We show that subsets of NPCs exhibit a unique connexin profile and that this profile is altered by laminin but not poly-L-lysine impacting upon functional channel activity.

## Results

### ECM effects on postnatal NPC culture

**Figure 1:**
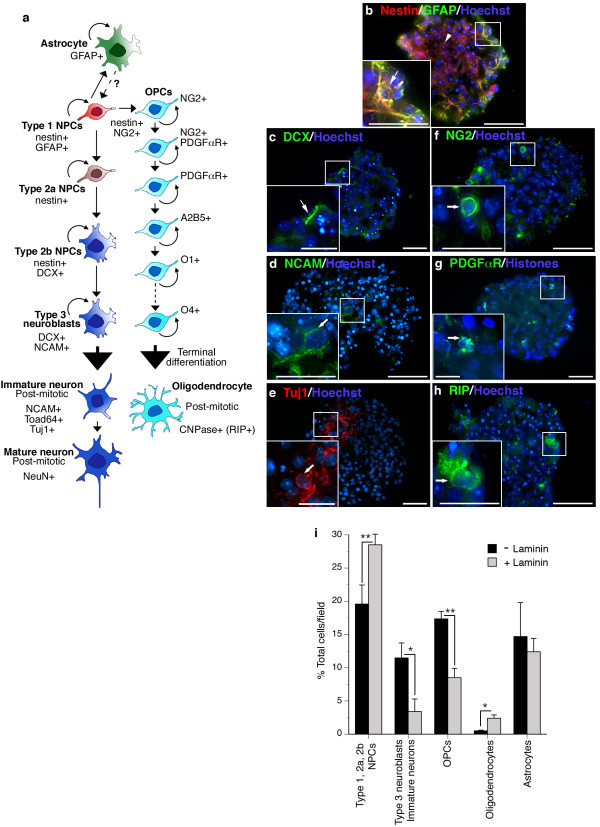
**Postnatal hippocampal-derived neurospheres are composed of subpopulations of progenitor and immature cell types**. Schematic of spontaneous specification over the course of neurosphere expansion *in vitro*. Pertinent lineage markers are indicated (a). Representative digital micrographs depicting antigenic lineage analysis of NPC populations through the central sections of serial cryosections of neurospheres cultured in the absence of laminin are depicted in b-h. Scale bars, 50 μm, insets 25 μm. Type 1 NPCs expressing GFAP and nestin localized to the periphery of spheres (b, arrows and inset); Type 2a NPCs expressing nestin were found toward the centre of the core (b, arrowhead). Type 3 neuroblasts (c, d, arrows and inset) and immature neurons (d, e, arrows and inset) were found in clusters throughout serial sections. NG2^+ ^(f, arrows and inset) and PDGFαR^+ ^(g, arrows and inset) OPCs and RIP^+ ^oligodendrocytes (h, arrows and inset) tended to be found along the neurosphere periphery. Culture on a laminin matrix increased the percentage of NPCs that retained a nestin^+ ^NPC identity and decreased the percentage of cells that specified to neuroblasts, immature neurons, and OPCs (i). Data represent mean of 5–15 sections counted over n = 5–10 cultures/condition ± standard error of measurement (SEM). *p < 0.05, **p < 0.01, ANOVA, *post-hoc *Tukey test.

To characterize neurosphere composition, we analysed marker expression in suspension and in cultures plated on a variety of adhesive substrates by immunofluorescence [see Additional file 1]. Although each neurosphere is likely derived from a single NPC, progeny spontaneously adopt distinct developmental lineages in culture (Fig 1a). We found neurospheres cultured in the absence of exogenous ECM were primarily composed of nestin^+ ^cells (Fig 1i). Cells co-expressing the Type 1 NPC marker GFAP localized to the periphery of neurospheres (Fig 1b). Type 2a cells expressing nestin only were found in the core of cultures (Fig 1b). DCX^+^, NCAM^+^, and TuJ1^+ ^neuroblasts and immature neurons were detected in clusters throughout the neurosphere structure (Fig 1c–e). NG2^+ ^and PDGFαR^+ ^oligodendrocyte progenitor cells (OPCs) and RIP^+ ^oligodendrocytes were found at the periphery of neurospheres with rare cells detected in the core (Fig 1f–h). When cultures were plated on laminin, we observed a significant increase in the percentage of cells that retained a nestin^+ ^NPC identity and a significant decrease in the percentage of cells that specified to neuroblasts, immature neurons, and/or OPCs (Fig 1i). No change in the number of astrocytes was detected but a small increase in RIP^+ ^oligodendrocytes was observed in laminin-plated cultures (Fig 1i).

### Intrinsic connexin mRNA expression

**Figure 2:**
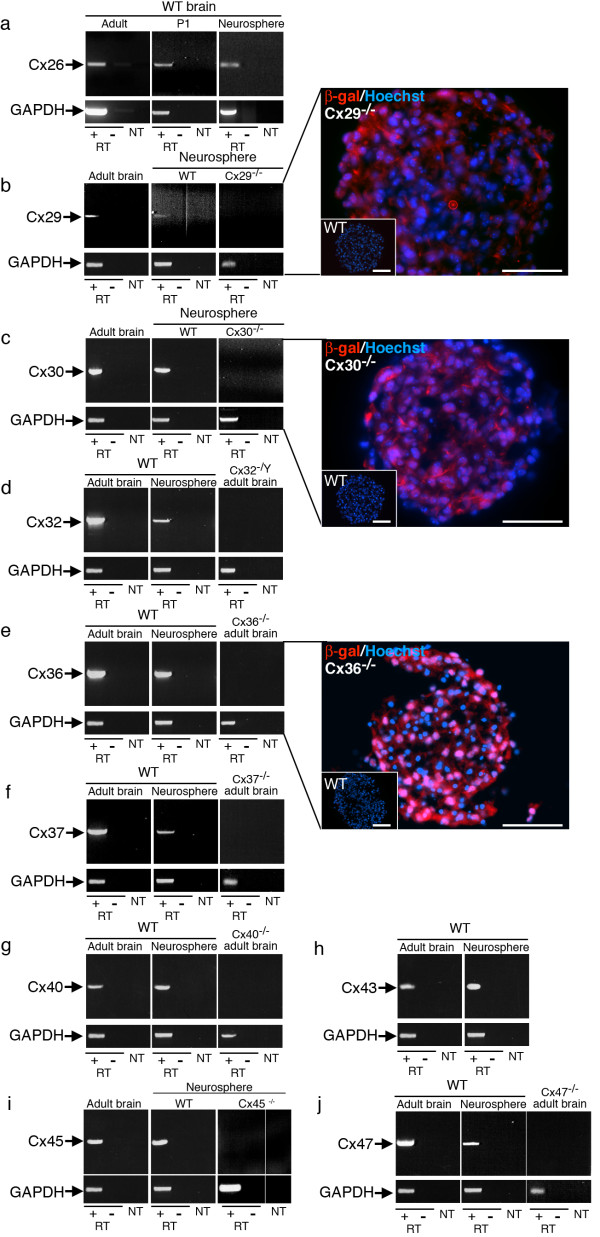
**Connexin mRNA expression in postnatal hippocampal neurospheres cultured in the absence of exogenous ECM**. RT-PCR analysis of Cx26 (a), Cx29 (b), Cx30 (c), Cx32 (d), Cx36 (e), Cx37 (f), Cx40 (g), Cx43 (h), Cx45 (i), and Cx47 (j) of random-primed RNA extracted from 100–150 pooled neurosphere cultures (+RT). GAPDH was amplified to confirm template integrity. Positive controls were: Total RNA isolated from adult and/or P1 WT whole brain. Negative controls included RT-PCR reactions performed on total RNA from null-mutant neurospheres (Cx29^-/-^, Cx30^-/-^, and Cx45^-/-^) or adult brain (Cx32^-/Y^, Cx36^-/-^, Cx40^-/-^, Cx37^-/-^, and Cx47^-/-^), and reactions processed in the absence of template (NT). Potential contamination by genomic DNA was assessed by omitting the RT enzyme from the reactions (-RT). In a, c, and e, panels depict neurosphere cryosections derived from Cx29^-/-^, Cx30^-/-^, and Cx36^-/- ^animals processed for β-galactosidase immunocytochemistry. The LacZ gene replaces the connexin coding sequence in each of these null-mutant animals. Insets are antibody controls demonstrating WT sections are negative for the β-galactosidase marker. Scale bars, 50 μm.

To identify the connexins expressed by postnatal progenitor cells in the absence of exogenous ECM we performed RT-PCR. Fourteen connexins (Cx26, Cx29, Cx30, Cx30.2, Cx31.1, Cx31.9, Cx32, Cx36, Cx37, Cx40, Cx43, Cx45, Cx47, Cx57) are expressed in the mammalian CNS [12-15]. Transcripts for ten of these connexins (Cx26, Cx29, Cx30, Cx32, Cx36, Cx37, Cx40, Cx43, Cx45, and Cx47) were detected in neurosphere culture (Fig 2a–j). Cx29, Cx30, and Cx36 expression was further confirmed by analysis of the reporter gene products present in null-mutant lines. In Cx29^-/- ^and Cx30^-/- ^mice, the lacZ open reading frame replaces the connexin coding sequences [20,21] whereas a bicistronic reporter cassette containing lacZ, an internal ribosome entry site, and placental alkaline phosphatase replaces the connexin coding sequence in Cx36^-/- ^mice [22]. β-galactosidase expression was readily detected in neurosphere cultures from these three null-mutant lines (Fig 2b, c, e).

### Differential expression of connexins in subsets of NPCs

**Figure 3:**
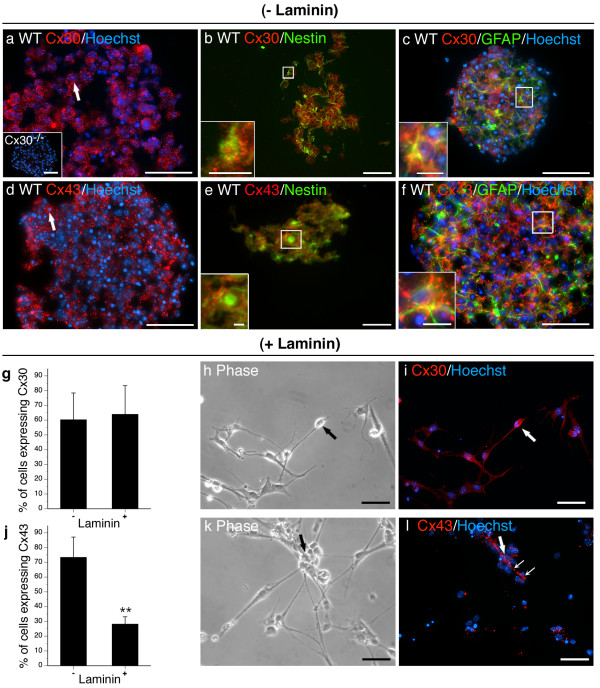
**Cx30 and Cx43 are expressed by Type 1 and Type 2a NPCs and are responsive to laminin**. Cx30 and Cx43 was detected in the majority of cells within neurospheres (a, d, arrows) and localized to cells expressing nestin (b, e, insets) and GFAP (c, f, insets). Inset in (a) demonstrates lack of Cx30 immunostaining on null-mutant neurospheres confirming antibody specificity. Adherence to laminin did not impact on the frequency of Cx30^+ ^cells (g) although a change in subcellular localization was observed (compare punctate membrane staining (a, arrows) with diffuse cytoplasmic staining (i, arrows)). A decrease in the frequency of Cx43^+ ^cells (arrows) was detected following culture on laminin (j). Cx43 was present at the plasma membrane between cells in direct apposition in the presence (a) or absence (b, small arrows) of laminin. Scale bars, 50 μm, insets in a, b, c, f, 25 μm, inset in e,10 μm. Data represent mean of 5–15 sections (- laminin) or field (+ laminin) counted over n = 5–10 cultures/condition ± SEM (g, j). **p < 0.01 Student's *t*-test.

**Figure 4:**
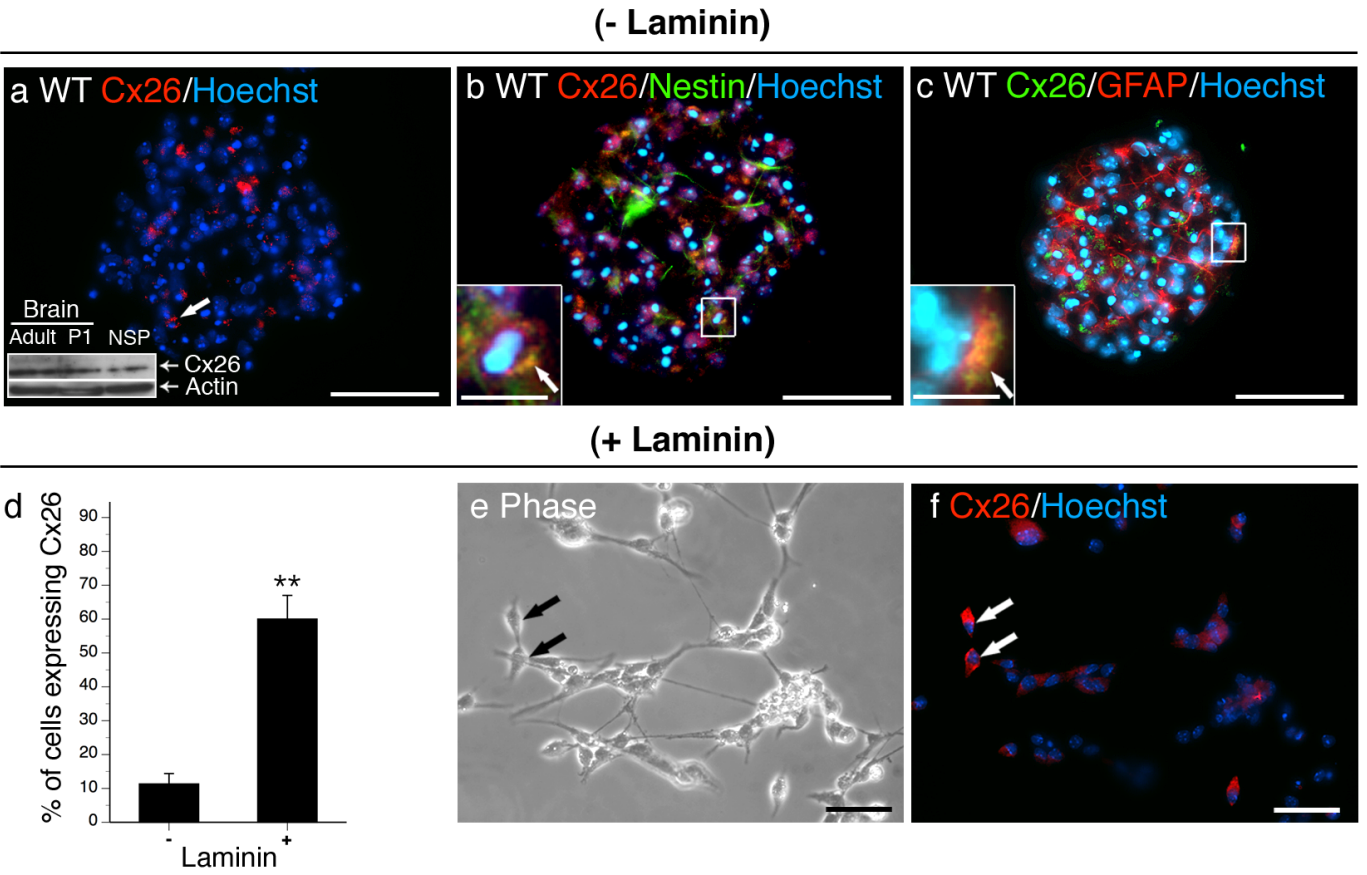
**Cx26 is expressed Type 1 and Type 2a NPCs and is responsive to laminin**. Cx26 was expressed by a small population of cells within neurospheres (a, arrow) and localizes to both cells expressing nestin (b, inset and arrow) and GFAP (c, inset and arrow). Inset in (a) depicts western blotting on protein derived from adult, P1 and neurospheres cultured in the absence of laminin to confirm antibody specificity. A marked increase in the frequency of Cx26^+ ^cells was detected when cultured on laminin (d, e, f, arrows). Data represent mean of 5–15 sections (- laminin) or field (+ laminin) counted over n = 5–10 cultures/condition ± SEM. (d). Scale bars, 50 μm, insets, 25 μm. **p < 0.01 Student's *t*-test.

**Figure 5:**
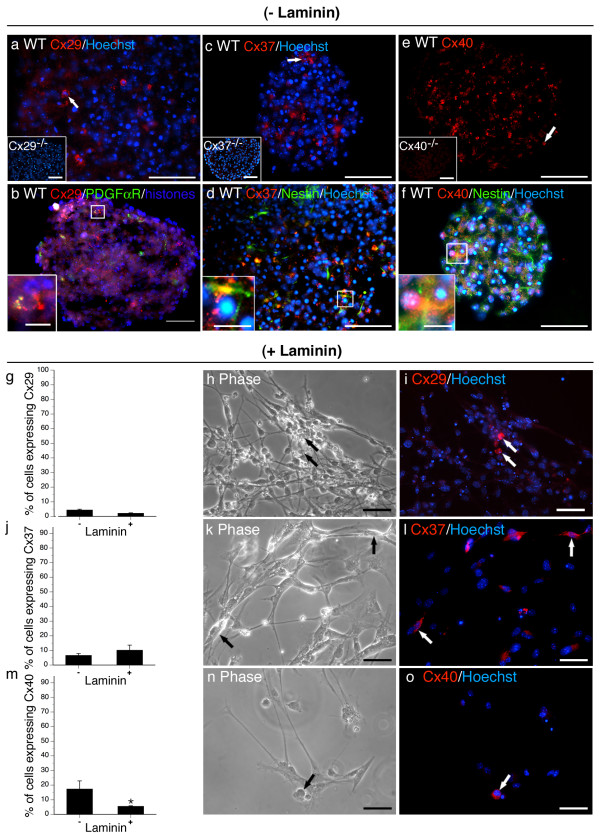
**Cx29, Cx37, and Cx40 are expressed by discrete cell populations with only Cx40 responsive to laminin**. Cx29 (a, arrow), Cx37 (c, arrow) and Cx40 (e, arrow) were found in a small number of cells within neurospheres. Insets depict immunostaining performed on null-mutant controls demonstrating antibody specificity. Cx29 was expressed by PDGFαR^+ ^OPCs (b). Cx37 and Cx40 expression were detected in nestin^+ ^Type 2a cells (d, f). Laminin did not alter the frequency of Cx29^+ ^(g-I, arrows) or Cx37^+ ^(j-l, arrows) cells but decreased the number of Cx40^+ ^cells detected in culture (m-o, arrows). Data represent mean of 5–15 sections (- laminin) or field (+ laminin) counted over n = 5–10 cultures/condition ± SEM (g, j.m). Scale bars, 50 μm. Inset in b, 10 μm. Inset in d, f, 25 μm. *p < 0.05 Student's *t*-test.

**Figure 6:**
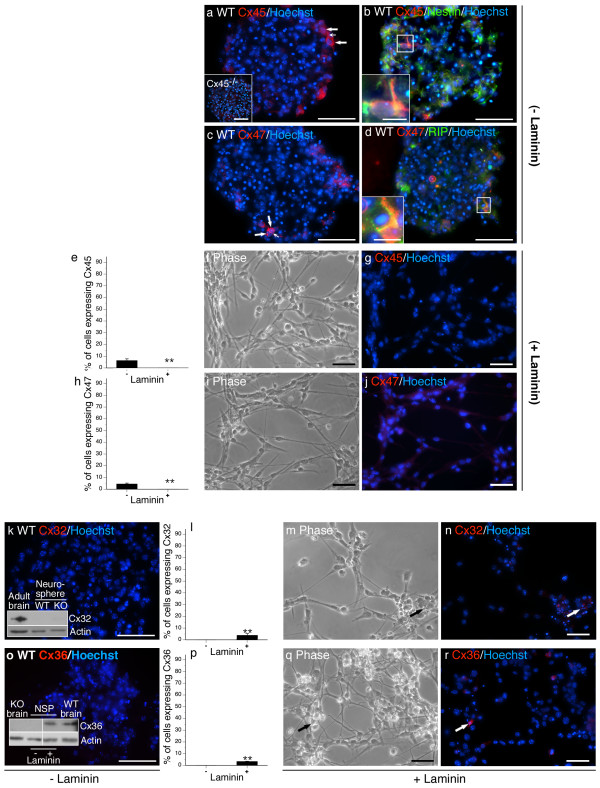
**Cx32, Cx36, Cx45, and Cx47 are expressed by rare cells and are laminin-responsive**. Cx45 (a, arrows) and Cx47 (c, arrows) were expressed in rare populations of cells. Cx45 was detected in Type 2a nestin^+ ^cells (b, inset). Cx47 was restricted to RIP^+ ^oligodendrocytes (d, inset). Both connexins were downregulated when plated on a laminin substrate (Cx45 e-g, Cx47 h-j). Cx32 (k) and Cx36 (o) protein was not detected in the absence of laminin but was induced following adherence to substrate (Cx32 (l-n) and Cx36 (p-r)). To confirm the lack of Cx32 protein in suspension cultures, western analysis was performed using adult WT brain as a positive control (k, inset). Similarly, western analysis was carried out to confirm Cx36 expression in the + laminin condition and to verify antibody specificity (p, inset). Adult WT and null-mutant (KO) brain served as positive and negative controls. Actin immunoblotting was performed as a loading control. Scale bars, 50 μm, Insets, 25 μm. **p < 0.01 Student's *t*-test.

We used immunofluorescence to localize connexins to NPC subtypes grown in the absence of exogenous laminin (Fig 3, 5, 6). For all connexins except Cx26, cultures from null-mutant mice served as negative controls to ensure antibody specificity. For Cx26, where deletion is embryonic lethal [23], antibody specificity was determined by Western blot (Fig 4a, inset). Four distinct connexin protein profiles were detected:

(I) Cx30 (Fig 3a) and Cx43 (Fig 3d) were expressed by the majority of cells (> 60%) throughout the structure of the sphere. Punctate membrane-associated immunoreactivity characteristic of gap junction labelling was evident in nestin^+ ^and GFAP^+ ^cells (Fig 3b–f). We used flow cytometry to quantify Cx30 expression in nestin^+^/GFAP^+ ^Type 1 NPCs, nestin^+ ^Type 2a NPCs, and GFAP^+ ^astrocytes [see Additional file 2]. We found that all of the Type 1 NPCs (93 ± 6%) and the vast majority of Type 2a NPCs (83 ± 2%) expressed Cx30. Surprisingly, few if any committed astrocytes were Cx30^+ ^(< 0.1%) [see Additional file 2]. These results were further substantiated by triple-labelling for Cx30, Cx43, and GFAP [see Additional file 3]. Cx43 but not Cx30 was detected in all GFAP^+ ^cell types suggesting ubiquitous localization of Cx43 in GFAP^+ ^NPCs and astroglia but a more restricted distribution of Cx30 to subsets of multipotential Type 1 GFAP^+ ^NPCs.

(II) Cx26 (Fig 4a), Cx29 (Fig 5a), Cx37 (Fig 5c), and Cx40 (Fig 5e) were expressed in a minority of cells (~10%) localizing to both the sphere core and periphery in the absence of laminin. Cx26 and Cx29 exhibited membrane-associated staining but rarely in apposition with neighbouring cells expressing the same connexin (Fig 4a, 5a arrows, 4b, 5b inset). Cx26 localized to nestin^+ ^and GFAP^+ ^Type 1 NPCs (Fig 4b, c) at the periphery of the sphere and nestin^+ ^Type 2a NPCs within the core (Fig 4b) as well as GFAP^+^/nestin^- ^astrocytes (Fig 4b, c). Cx29 localized to PDGFαR^+ ^OPCs (Fig 5b, inset). Cx37 and Cx40 labelling appeared to be largely intracellular (Fig 5c, e, arrows) suggesting these connexins are not forming gap junction channels at the plasma membrane. Both Cx37 and Cx40 were expressed primarily by nestin^+ ^Type 2a cells (Fig 5d, f, insets).

(III) Cx45 (Fig 6a) and Cx47 (Fig 6c) were expressed by rare cells (< 10%) restricted primarily to the periphery of neurospheres cultured in the absence of laminin. These connexins were often expressed in adjoining cells (Fig 6a, c, large arrows) where contiguous labelling was detected at the plasma membrane (Fig 6a, c, small arrows). Cx45 localized to nestin^+ ^Type 2a NPCs (Fig 6b, inset) and was not detected in GFAP^+ ^cells (data not shown). Cx47 expression was restricted to RIP^+ ^oligodendrocytes (Fig 6d, inset).

(IV) Both Cx32 and Cx36 were detected at the mRNA level (Fig 2d, e) but no immunodetection of protein was observed (Fig 6k, o). Antibody efficacy was confirmed by Western analysis (Fig 6k, o, insets).

### Exposure to exogenous laminin substrate alters NPC expression of most connexins

To assess the effect of ECM on connexin expression, NPCs were expanded for 8 DIV in suspension before plating on laminin-coated glass coverslips [see Additional file 1]. Cultures exposed to laminin rapidly adhered to the ECM substrate. Cells at the periphery of the neurosphere migrated from the core as adherent monolayers over 6 DIV. Fewer Cx43^+ ^(Fig 3j–l) and Cx40^+ ^cells (Fig 5m–o) and no Cx45^+ ^(Fig 6e–g) or Cx47^+ ^(Fig 6h–j) cells were observed when NPCs were cultured on laminin substrate. Laminin increased the number of Cx26^+ ^cells (Fig 4d–f) and induced Cx32 (Fig 6l–n) and Cx36 (Fig 6p–r) protein. Cx32^+ ^cells exhibited small nuclei (Fig 6m, n, arrow) and were immunopositive for the OPC marker NG2 (data not shown). Cx36 was expressed in refractile cells with neuritic extensions (Fig 6r, s, arrow) and were immunoreactive for the neuronal marker Tuj1-βIII tubulin (data not shown). Although exposure to the ECM substrate did not alter the percentage of cells expressing Cx30 (Fig 3g), a change in subcellular localization was observed. Immunoreactivity was markedly less punctate (compare Fig 3a and 3i) with protein detected throughout the cytosol of laminin-treated cultures (Fig 3h, i, arrows). Contact with laminin did not change the number of cells expressing Cx29 (Fig 5g–i), Cx30 (Fig 3g–i), or Cx37 (Fig 5j–l).

**Figure 7:**
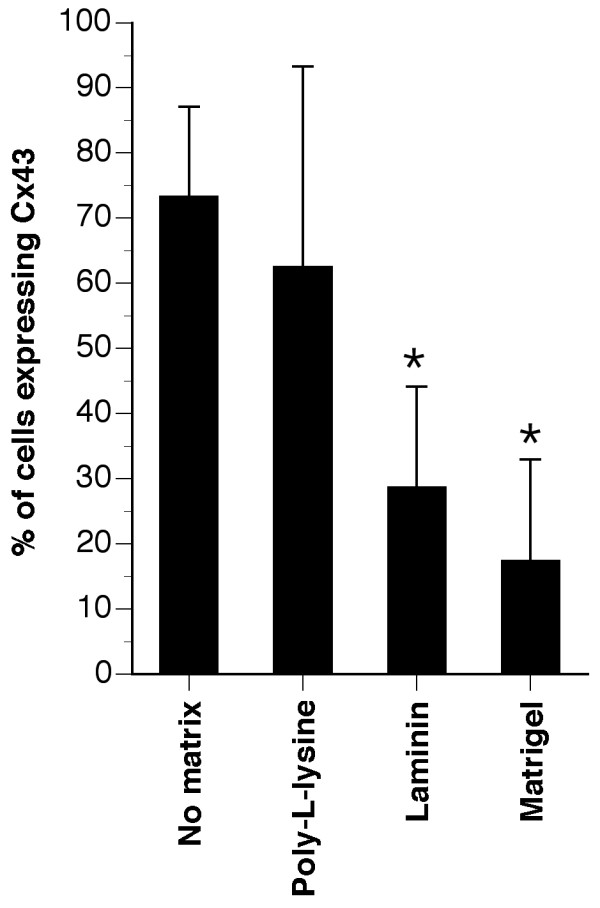
**Laminin and laminin-ECM mixtures but not poly-L-lysine alter the expression of Cx43**. The number of Cx43 expressing cells was reduced when NPCs were plated on laminin or a Matrigel matrix composed of laminin/collagen/entactin but not when cultures were plated on poly-L-lysine. *p < 0.05, ANOVA, *post-hoc *Dunnett's *t*-test compared to neurospheres cultured in the absence of matrix.

An alternative explanation for these changes lies in the mechanical impact of NPC plating to an adhesive substrate [16,24,25]. To test this, we plated neurospheres on poly-L-lysine, laminin, or a Matrigel mixture containing the following ECM components (56% laminin, 31% collagen IV, 8% entactin) and assessed Cx43 expression (Fig 7). The same reduction in Cx43-expressing cells was observed following engagement with Matrigel as plating on laminin alone. Plating on poly-L-lysine had no effect on Cx43 expression. Taken together, these data provide converging evidence for a laminin-specific regulation of connexin expression as compared to a physico-mechanical influence of culture condition.

### Impact of laminin-induced changes in connexin expression on cell-cell and hemichannel communication

**Figure 8:**
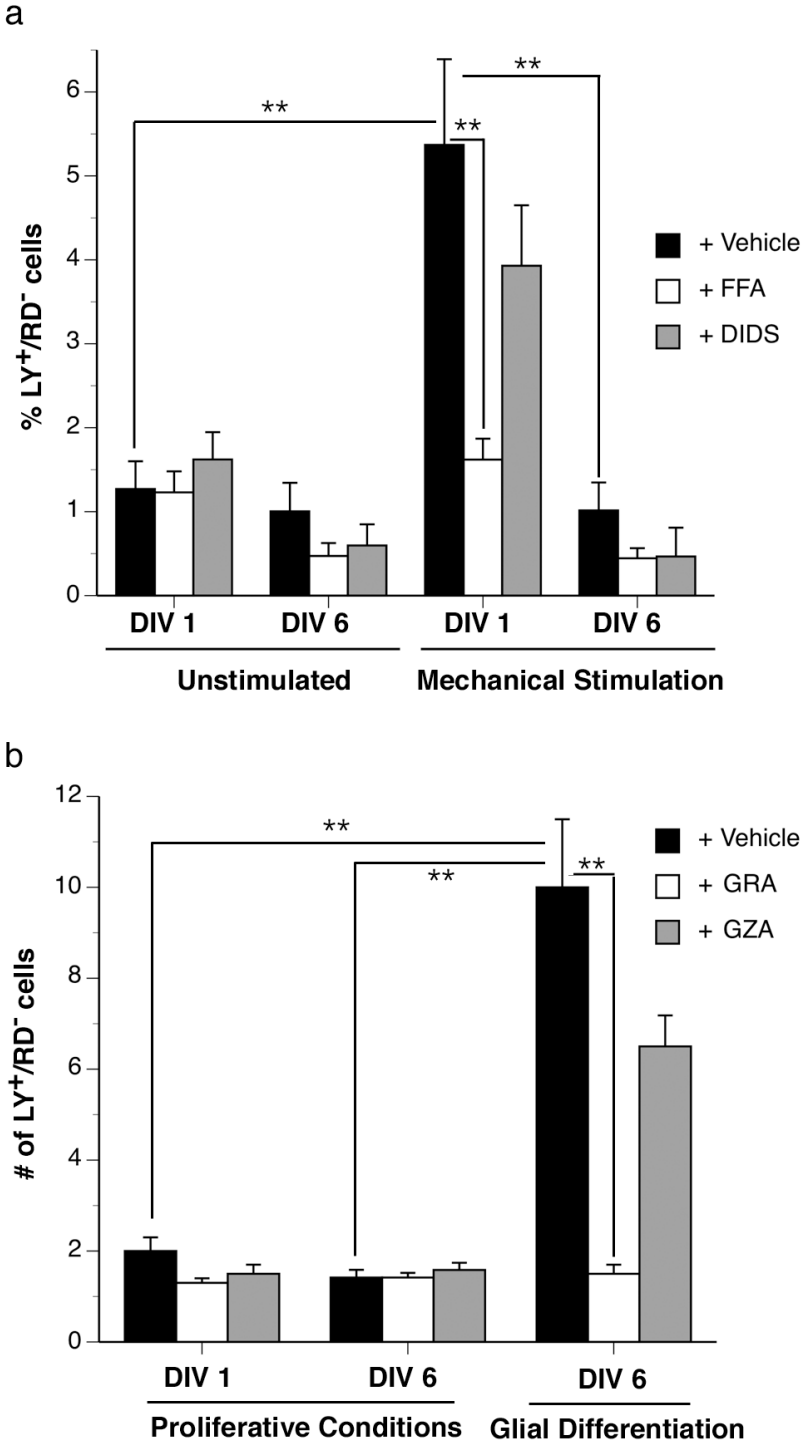
**Laminin engagement alters functional hemichannel but not GJIC activity**. Functional hemichannel activity was assessed by anionic LY dye uptake (a) of cultures exposed to laminin for 1 DIV (prior to any changes in connexin expression) or 6 DIV (following changes in connexin expression). Spontaneous LY uptake was observed at low levels after 1 and 6 DIV. Open hemichannel activity could be induced by mechanical stimulation with glass microbeads within 1 DIV but not 6 DIV. Dye uptake was inhibited by the hemichannel/chloride channel inhibitor FFA but not the chloride channel blocker DIDS. LY^+^/RD^- ^cells is expressed as a percentage of the total number of cells per microscopic field ± standard error of the mean (SEM) counted in n = 5 fields per experiment conducted in triplicate experiments. GJIC was assayed using the scrape-loading method (b). Significant LY^+^/RD^- ^dye transfer was not observed after 1 or 6 DIV when NPCs were cultured in the presence of mitogens (proliferative conditions). As a positive control, we performed the same experiment in a condition known to promote glial cell differentiation and obtained robust GJIC that was significantly inhibited by the gap junction blocker GRA but not the inactive analog GZA (glial differentiation). In GJIC assays, the number of LY^+^/RD^- ^cells along the scrape line was established in serial photographs taken along the entire length of the scrape (9–14 photos/coverslip) over triplicate cultures. Two coverslips were assessed per culture for a total of n = 54–84 measurements per condition. Data are expressed as the mean number of LY^+^/RD^- ^cells ± SEM. *p < 0.05, **p < 0.01, ANOVA, *post-hoc *Tukey test.

Transmembrane flux of the low molecular mass fluorescent dye, LY, was used to assess hemichannel activity in neurosphere cultures. RD was used to control for uptake resulting from plasma membrane damage. Hemichannel opening was induced by mechanical stimulation with glass microbeads as previously described [26]. Cultures were expanded in suspension for 8 DIV, then plated and analyzed immediately after adherence (Fig 8a, DIV 1) prior to any detectable change in connexin protein expression (data not shown) or after 6 DIV in contact with laminin (Fig 8a, DIV 6) following the observed changes in connexin expression (Fig 3, 4, 5). Mechanical stimulation elicited a significant increase in dye uptake on DIV 1 (Fig 8a, DIV 1) that was largely inhibited by the dual-specificity chloride channel and connexin/pannexin-channel blocker FFA [27,28] but not the chloride channel inhibitor DIDS (Fig 8a, DIV 1). Hemichannel activity was lost when NPCs were cultured on laminin for 6 DIV (Fig 8a, DIV 6). We cannot, however, rule out that these changes are due to an effect of ECM on pannexin channel formation as we have determined that NPCs cultured in suspension express both pannexin 1 and 2 mRNA (data not shown) yet we have not investigated impact of laminin on this expression at the protein level.

Biochemical coupling indicative of GJIC was assessed by scrape loading. Little to no dye transfer was observed on DIV 1 or DIV 6 (Fig 8b, Proliferative conditions, DIV 1 and DIV 6,). These data were surprising given the ubiquitous Cx30 and Cx43 expression detected in the majority of cells prior to laminin engagement. As a positive control, we differentiated NPCs to a predominantly nestin^-^/GFAP^+ ^astrocytic lineage [see Additional file 4]. Robust GJIC was detected in these cultures and was inhibited by gap junction channel blocker GRA but not its inactive analog GZA (Fig 8b). To provide mechanistic insight into the lack of LY transfer in NPC cultures, we assessed Cx43 phosphorylation status and found, in neurospheres cultured without laminin, that the P2, P3, and hyperphosphorylated forms of Cx43 predominated [see Additional file 5]. Previous studies have indicated that the degree of LY dye coupling is inversely correlated with phosphorylation state [29] suggesting that post-translation modification of Cx43 in NPC cultures may account for this lack of dye transfer. Taken together, these data indicate that postnatal NPC cultures do not establish gap junctions permeable to LY, but do exhibit hemichannel activity that is suppressed upon exposure to laminin.

## Discussion

Our data indicate hippocampal-derived postnatal progenitor cells express a broad array of connexin genes and that connexin expression, intracellular distribution, and channel activity are regulated by ECM-cell interactions. When cultured as neurospheres in the absence of exogenous ECM, postnatal NPCs express Cx26, Cx29, Cx30, Cx37, Cx40, Cx43, Cx45, and/or Cx47 mRNA and protein as well as Cx32 and/or Cx36 mRNA. Exposure to a laminin substrate markedly increases the number of Cx26^+ ^cells present in culture and elicits the appearance of Cx32 and Cx36 protein. Conversely, laminin treatment decreases the frequency of Cx40^+^, Cx43^+^, and Cx45^+ ^cells without impacting on the number of Cx29^+^, Cx30^+^, or Cx37^+ ^NPCs. Surprisingly, we found that, while postnatal NPC cultures exhibit hemichannel activity, they do not transfer LY, a well-established hallmark of junctional communication, until differentiated to a mature astroglial lineage. The lack of dye transfer is likely the result of post-translational modification of the connexin proteins, specifically Cx43. Further, hemichannel activity is suppressed by plating on laminin. Together, these findings suggest a new role for ECM-cell interaction, specifically laminin, in the regulation of intrinsic connexin expression and function in postnatal NPC cultures. This regulation may predispose postnatal NPCs toward channel-independent connexin communication as recently demonstrated in the developing CNS wherein channel-independent connexin adhesion influences the migration of newly born neurons along radial glia [7].

### Localization of connexins to discrete subsets of postnatal hippocampal progenitor cells

The repertoire of connexins identified here is much larger than that reported by Wörsdöfer *et al*., [30] who demonstrated expression of seven connexin transcripts but protein for only Cx31, Cx43, and Cx45. However, these studies used ECM components and not suspension-grown neurospheres. Furthermore, they used embryonic stem (ES) cells rather than postnatal NPCs. Thus, the differences in connexin expression patterns likely reflect the unique connexin signature of the different stem and progenitor cell populations present in these cultures as well as the impact of ECM adhesion on connexin expression. Here, we show that Type 1 and Type 2a multipotential NPCs express Cx26, Cx30, and Cx43. Cx26 and Cx43 have been previously observed in embryonic NPC populations [31,32] but this is the first demonstration that Cx30, exclusively expressed postnatally [8,33], is also expressed by multipotential Type 1 NPCs. We found that progression to a Type 2a lineage is associated with *de novo *Cx37, Cx40, and Cx45 protein expression. Further commitment to an oligodendrocyte lineage is accompanied by the loss of all of the connexins identified in multipotential NPCs and *de novo *expression of connexins associated with mature oligodendrocytes (Cx29, Cx32, Cx47) [34]. Surprisingly, Cx32 transcript but not protein was detected in OPCs and oligodendroglia in the absence of laminin. The presence of Cx32 transcript but not protein is also observed in ES cells [30] with unknown consequence on cell fate. Taken together, these data provide evidence of intrinsic connexin signatures expressed by progenitor cell subsets over the course of commitment *in vitro*.

### Effect of laminin on connexin expression and function

Exposure of NPCs to a laminin substrate altered connexin protein expression and/or stability, intracellular distribution, and functional channel activity. The simplest explanation for these changes is that this profile mirrors the effect of laminin on the differentiation of NPCs. Plating of NPCs on ECM is known to alter the rate of spontaneous specification and survival of embryonic stem cell cultures [35-37]. Indeed, we found more cells retained a multipotential Type 1/2 phenotype in laminin-treated cultures than those expanded in the absence of laminin. An alternative explanation is a direct effect of laminin/integrin signalling on the regulation of connexin protein expression or stability in NPCs. Certainly, laminin has been shown to alter connexin expression and function in other cell types [17-19]. The consequence of these changes have only begun to be appreciated. In CNS, the recent finding that gap junctional plaques formed by Cx26 or Cx43 are required for migration of neuronal progenitors along radial glial cells during cortical development in the mouse [7] suggests that ECM regulation of connexin expression is involved in cortical lamination and thus is likely to influence postnatal NPC migration. This hypothesis represents an unexplored role for ECM-connexin interactions *in vivo*.

The changes in connexin profile detected in the presence of laminin are not restricted to NPCs. OPC and neuronal profiles are also altered. Cx32 and Cx36 protein, not observed in the absence of exogenous ECM, are present following laminin engagement. Cx32 null-mutation has previously been shown to delay terminal differentiation of NG2^+ ^OPCs in adult hippocampus [38]. Here, we find that the induction of Cx32 protein in NG2^+ ^OPCs by laminin is associated with a moderate increase in the frequency of RIP^+ ^oligodendrocytes suggesting enhanced oligodendrogenesis. Surprisingly, these new oligodendrocytes did not express Cx47 in the presence of laminin suggesting that perhaps different functional subset of cells are generated. Recent studies indicate that Cx32 and Cx47 do not oligomerize and do not form the same heterotypic intercellular channels taken as evidence for functional differences between these connexins and perhaps between subsets of oligodendrocytes [39,40]. Finally, the induction of Cx36 protein in TuJ1^+ ^neurons in the face of reduced neurogenesis was equally surprising. This observation may recapitulate the changes observed between early and late phases of embryonic neurogenesis. Cx36 mRNA is initially detected in multipotential neural stem cells during early but not late neurogenesis where expression is restricted to their neuronal progeny [8,41]. It may be that the functional changes in channel activity observed following engagement of neurosphere cultures with laminin regulate NPC and OPC commitment.

At no point did we detect cell-cell coupling with respect to the passage of the anionic dye LY. While surprising given that embryonic progenitor populations are coupled during early neurogenesis [42-44] our data agree with the findings of Duval *et al. *who demonstrated GJIC in astrocytes but not in other NPC-derived cells [45]. Here, we show passage of LY only when NPC cultures are induced to differentiate towards a primarily astroglial lineage and likely corresponds with a shift in the phosphorylation status of Cx43. Conversely, robust transmembrane dye flux indicative of functional hemichannel activity is evident prior to ECM-induced changes in connexin protein profiles. Laminin engagement suppresses this activity. It is tempting to speculate that connexin (or pannexin)-mediated hemichannel activity promotes specification whereas channel-independent adhesion supports the migration through ECM-defined domains in postnatal hippocampus. However, care must be taken in directly extrapolating our results to the *in vivo *situation given the complex three dimensional character of the developing hippocampus where many different matrices and cellular scaffolds are present given evidence of different cell phenotypes within 2D and 3D culture systems [16]. Clearly, it will be important to establish the impact of ECM control of connexin protein expression on postnatal NPC proliferation, migration, and specification *in vivo*.

## Conclusion

We have identified the repertoire of connexins expressed by postnatal hippocampal NPCs over the course of commitment *in vivo*. We have demonstrated that ECM engagement, more specifically laminin, can alter not only the fate of cultured postnatal hippocampal NPCs, but also their connexin expression profile and related channel function. Taken together, these data suggest that ECM control of connexin expression and signalling may play a role in the direction of multipotential NPCs towards a neuronal or glial lineage.

## Methods

### Mice

Breeding pairs of Cx29^-/-^, Cx36^-/-^, Cx37^-/+^, Cx40^-/-^, Cx45^F/F^, and Cx47^-/- ^animals were generated by our laboratories as described [22,34,46-49]. Cx32^-/- ^and Cx30^-/- ^animals [20,50] were kindly provided by Dr. Klaus Willecke (University of Bonn). Nestin-cre recombinase transgenics [51] were kindly provided by Dr. Ruth Slack (University of Ottawa). Each strain was backbred for 3–12 generations into a C57BL/6 lineage. All null-mutant mice were compared to congenic wild-type (WT) littermates. Mice were kept on a 12 h light-dark cycle and allowed food and water *ad libitum*. Mice were genotyped using the primer pairs listed in [see Additional file 6]. All experimental protocols were approved by the Animal Care Committee of the University of Ottawa according to guidelines set forth by the Canadian Council on Animal Care.

### Neurosphere suspension culture

All chemical reagents were obtained from Sigma-Aldrich (St.-Louis, MO, USA) and all cell culture reagents were obtained from Invitrogen (Burlington, ON, Canada) unless otherwise stated. NPCs were cultured as described in [52] with some modifications. Briefly, cells were isolated from the hippocampi of postnatal day 0 – 2 (P0–P2) mouse pups. Animals were sacrificed by lethal injection with Euthansol (Schering-Plough Canada Inc., Pointe-Claire, QC, Canada) and 500 μm sections between bregma -1.7 mm and -2.2 mm were prepared on a VT1000S vibratome (Leica Microsystems Inc.) in ice-cold artificial cerebral spinal fluid (26 mM NaHCO_3_, 124 mM NaCl, 5 mM KCl, 2 mM CaCl_2_, 1.3 mM MgCl_2_, 10 mM D-glucose, 100 U/ml penicillin, and 100 μg/ml streptomycin). Hippocampi from n = 2–6 pups were pooled for each culture. Under a Leica MZ6 dissecting microscope, hippocampi were removed and dissected free of blood vessels and choroid plexus. Hippocampi were minced with a scalpel and enzymatically dissociated in: 26 mM NaHCO_3_, 124 mM NaCl, 5 mM KCl, 0.1 mM CaCl_2_, 3.2 mM MgCl_2_, 10 mM D-glucose, 1% penicillin/streptomycin, 0.1% neural protease, 0.01% papain, and 0.01% DNase I for 45 minutes at 37°C. Single cells were resuspended in expansion media (Dulbecco's modified Eagle's medium F12 (DMEM/F12), 2 mM L-glutamine, 100 U/ml penicillin, 100 μg/ml streptomycin, 1× B27 supplement, 20 ng/ml human recombinant epidermal growth factor (EGF), and 10 ng/ml basic fibroblast growth factor (FGF-2)) in 60 mm Petri dishes (Fisher Scientific, Nepean, ON, Canada). Cell viability was established by Trypan Blue hemacytometer counts and cells plated at a density of 2.5 × 10^5 ^viable cells/dish. This protocol typically yields 200 viable neurospheres/60 mm dish cultured in suspension. Cultures were maintained at 37°C in a 5% CO_2 _atmosphere with fresh EGF and FGF-2 added every two days for 8 days *in vitro *(DIV).

### Laminin, Matrigel, and Poly-L-Lysine Treatment

On DIV 8, neurospheres were plated in 10 cm tissue culture dishes containing laminin (15 μg/mL), Matrigel (Invitrogen), or poly-L-lysine (100 μg/ml)-coated glass coverslips (Corning, NY, USA) and cultured in expansion media supplemented with EGF and FGF-2 or in differentiation media: DMEM/F12, 2 mM L-glutamine, 1 mM sodium pyruvate, 200 mM D-glucose, 100 U/ml penicillin, 100 μg/ml streptomycin, and 1× N2 supplement containing EGF and FGF-2 or 0.5 μM retinoic acid and 0.5% fetal bovine serum. The latter condition promotes astroglial specification [53]. Cells were not dissociated to maintain the 3D topography of the cultures. Neurospheres adhered to each substrate and began to grow as monolayer colonies within 24 h of transfer. To maintain NPC proliferation, half the volume of media was removed and replenished every 2 days with fresh addition of EGF and FGF-2 to final concentrations of 10 and 20 ng/ml for 6 DIV (total 14 DIV). Some cultures were pulsed with 20 μg/mL 5'-bromo-2-deoxyuridine (BrdU, Roche Diagnostics, Laval, QC) 24 h prior to plating on laminin to determine the percentage of actively dividing cells in suspension that generated mature progeny. Cultures exposed to exogenous matrix or adhesive substrate were compared to cultures maintained in suspension in the same media supplemented with EGF and FGF-2. All immunocytochemistry and western analysis were performed on DIV 14.

### Reverse transcriptase-polymerase chain reaction (RT-PCR)

Total RNA was isolated from WT, Cx29^-/-^, Cx30^-/-^, and Cx45^-/-^neurospheres and from adult and/P2 WT, Cx32^-/-^, Cx36^-/-^, Cx37^-/-^, Cx40^-/-^, and Cx47^-/- ^mouse brain using Trizol Reagent (Invitrogen). RNA was collected from one dish per experiment. Each assessment was confirmed in duplicate or triplicate. Total RNA was treated with DNaseI (Promega, Madison, WI, USA). First-strand synthesis was performed using pdN_6 _(Promega) random primers and Superscript II RT (BD Biosciences, San Jose, CA, USA) according to manufacturer's recommendations. PCR was performed using the primers and conditions listed in [see Additional file 6]. The PCR reaction contained, in a final volume of 25 μl: 1× PCR buffer, 0.8 mM dNTPs, 1× Advantage 2 Polymerase (Clontech, Cambridge, ON, Canada). All reactions were carried out using the following cycling parameters: 94°C for 5 min, 35 cycles of 94°C for 25 sec, 59°C for 50 sec, and 72°C for 1 min 45 sec, and a final step at 72°C for 7 min in a Whatman Biometra T-Gradient thermocycler (Montreal-Biotech Inc., Kirkland, QC, Canada).

### Western analysis

Neurospheres were washed in PBS (10 mM PBS: 0.154 M NaCl, 0.0028 M NaH_2_PO_4_, 0.0072 M Na_2_HPO_4_, pH 7.2), pelleted, and resuspended in RIPA buffer (1% Nonidet P-40, 0.5% sodium deoxycholate, 0.1% sodium dodecyl sulphate (SDS), 1 mM sodium fluoride, 1 mM sodium orthovanadate, 50 μg/ml aprotinin, and 1 mg/mL phenylmethylsulphonylfluoride in PBS). Where Western analysis of cultures grown on laminin substrate is indicated, adherent cultures were washed with PBS and RIPA buffer added directly to plates. Cells were collected using a cell scraper (Fisher), incubated on ice for 30 min, then centrifuged at 13 400 × g for 30 min. Protein was isolated from mouse brain homogenized in RIPA buffer using a Tissue Tearor (Biospec Products Inc., Bartlesville, OK, USA). Protein concentration was determined using the Bio-Rad DC protein assay kit (Bio-Rad, Hercules, CA, USA) according to manufacturer's protocol. Protein samples of 30 μg were separated by SDS-polyacrylamide gel electrophoresis and transferred to polyvinylidene difluoride membranes (Fisher). Membranes were blocked at room temperature for 1 h with 1% casein in PBS. Primary and secondary antibodies are listed in [see Additional file 6]. Immunoreactivity was visualized using the SuperSignal West Pico Chemilunescent Substrate kit (Pierce Biotechnology Inc., Rockford, IL, USA). All assessments were performed in duplicate.

### Immunocytochemistry

Neurospheres expanded in suspension or grown on laminin were fixed in 3.7% formaldehyde solution in PBS for 20 min. Following extensive washes in PBS, neurospheres were cryoprotected for 24 h with 15% sucrose in PBS + 0.001% NaN_3_, flash-frozen, and serially sectioned (10 μm thickness) using a cryostat (Leica CM1900), Immunocytochemistry was carried out as previously described [38]. Antibodies are listed in [see Additional file 6] and were diluted in 3% bovine serum albumin + 0.3% Triton X-100 in PBS. Hoechst 33258 (1 μg/mL) was used as a nuclear counterstain. For suspension cells, the number of connexin^+ ^cells was expressed as the percentage of Hoechst^+ ^nuclei counted in serial sections of 10–50 neurospheres. For cultures plated on laminin, immunocytochemistry was performed directly on laminin-coated glass-coverslips. Connexin^+ ^cells/Hoechst^+ ^nuclei were established in five fields per neurosphere calculated for 5–10 neurospheres per connexin (n = 25–50 fields per condition). Because cell density in the sphere core was such that individual cells could not be quantified or verified to be in contact with the laminin substrate [see Additional file 7, area outlined in black], cell counts were performed over five defined peripheral cell fields shot at 40× magnification [see Additional file 7]. All cell images were taken with a Leica DMXRA2 epifluorescence microscope and analyzed using Openlab software v5.05 (Improvision, Lexington, MA, USA).

### Flow Cytometry

Cultures were expanded as neurospheres for 14 DIV before being suspended in 2% formaldehyde in 10 mM PBS and triturated to achieve a single cell suspension. Following two PBS washes and gentle centrifugation at 2000 rpm for 5 min, NPCs were suspended in PFN (2% fetal bovine serum, 0.1% NaN_3_, 0.18% saponin in 10 mM PBS). Cell suspensions were separated into 1.5 ml microfuge tubes at a concentration of 1 × 10^6 ^cells per tube. Primary or isotype control antibodies were added directly to cell suspensions at appropriate concentrations followed by incubation at room temperature for 30 min on a shaker. Each sample was washed twice by the addition of 0.8 ml of PFN and gentle centrifugation. Secondary antibodies were added to cell suspensions at appropriate concentrations followed by incubation at room temperature for 30 min on a shaker and washed as described above. Cells were resuspended in 0.5 ml of PFN and analyzed using a Beckman Coulter FC500 Flow Cytometer (Beckman Coulter Canada Inc., Mississauga, ON) and Beckman Coulter CXP software.

### GJIC and hemichannel assay

Dye uptake and dye transfer assays were assessed on 10 neurospheres/condition in two separate experiments per condition. For quantification, four fields were photographed per sphere. For dye coupling assays, photomicrographs represented two fields within the core of the neurosphere and two fields at the periphery where cells were in direct apposition and thus potentially coupled. To image cells within the core in direct contact with matrix, micrographs were obtained using a Leica DMIR epifluorescent inverted microscope equipped with a QICAM digital camera (Quorum Technologies, Guelph, ON, Canada) and captured using OpenLab software v5.05. For hemichannel assays, all four fields were shot along the periphery and were taken with a Leica DMXRA2 epifluorescence microscope and analyzed using Openlab software (v5.05). Cultures expanded in suspension as neurospheres for 8 DIV were plated on coverslips in expansion media containing (a) EGF and FGF-2 for 1 DIV to assess function prior to laminin-induced changes in connexin expression, (b) EGF and FGF-2 for 6 DIV to assess connexin function after laminin-induced changes, or (c) in differentiation media for 6 DIV supplemented with RA (0.5 μM) and FBS (0.5%) to promote glial differentiation. Hemichannel activity was assessed by comparing uptake of connexin channel-permeant Lucifer yellow (LY, 457 Da) and channel-impermeant rhodamine B isothiocyanate-dextran (RD, 10,000 Da) in the presence or absence of 50 μM flufenamic acid (FFA) or 100 μM 4,4'-diisothiocyanatostilbene-2,2'-disulfonic acid (DIDS) as previously described [26]. Mechanical stimulation with glass microbeads was used to trigger hemichannel opening [26,28]. Cells positive for both LY and RD were excluded from the measurements to control for LY uptake resulting from loss of membrane integrity. For all assays, the number of LY^+^/RD^- ^cells is expressed as a percentage of the total number of cells per microscopic field ± standard error of the mean (SEM). Dye transfer between cells indicative of GJIC was assessed by scrape loading in the presence or absence of the intercellular channel blocker 18α-glycyrrhetinic acid (GRA, 100 μM) or its inactive analog glycyrrhizic acid (GZA, 100 μM) as previously described [26]. Dye transfer was quantified by counting the number of LY^+^/RD^- ^cells emanating from a LY^+^/RD^+ ^cell adjacent to the scrape line. Data are expressed as the mean number of LY^+^/RD^- ^cells ± SEM.

### Statistics

Data were analyzed by analysis of variance (ANOVA) followed by *post hoc *Tukey tests or Student's *t *test where appropriate.

## Authors' contributions

SI and LGG performed the majority of the experimental work. SI, LGG, and SALB conceived and designed the experiments, analyzed the data, interpreted the results, and prepared the figures. CDS and RD carried out and interpreted the quantification of the lineage analysis with SI. CDS performed the immunogenic analyses of Cx36-/- neurospheres. LM participated in and interpreted the functional communication assays. AP performed the Cx45 RT-PCR and the comparison of different matrices on Cx43 expression. HDT participated in the Cx30 immunoassays and performed all of the flow cytometry experiments. ASM, KN, DLP, and AMS created and provided key study material and assisted in data analysis. DLP and AMS helped draft the manuscript. SI and SALB wrote the manuscript. SALB coordinated the study and provided financial support. All authors read and approved the final manuscript.

## Supplementary Material

Additional file 1 **Schematic representation of the neurosphere culture protocol and analysis.** A graphic depiction of the protocol employed to culture and analyse neurospheres in the presence and absence of laminin. Click here for file

Additional file 2 **Cx30 is expressed by Type 1 and 2a NPCs.** (a) Hippocampal NPCs cultured as neurospheres were dissociated on DIV 14 and triple-labelled for Cx30, nestin, and GFAP. Two and three dimensional histograms from a representative flow cytometry analysis are presented for both isotype control (left panel) and experimental (right panel) reactions. The quadrant percentages represent the percentage of total cells expressing corresponding cell markers. (b) Quantitative analysis of Cx30 expression in Type 1 (GFAP+/nestin+) NPCs, Type 2a (GFAP-/nestin+) NPCs, and committed astrocytes (GFAP+/nestin-). The percentage of cells co-expressing Cx30 was calculated divided by the total percentage of each cell type by the total percentage of cells within each cell type expressing Cx30 in triple-labelling studies. Data are presented as mean ± SEM and represent the average of three independent flow cytometric analyses/cultures. Click here for file

Additional file 3 **Cx43 but not Cx30 is detected in all GFAP^+ ^cell types.** A 10 μm cyrosection through a neurosphere harvested on DIV 14 triple-labelled for Cx30, Cx43, and GFAP is presented. Yellow arrow points to cells expressing GFAP/Cx30/ and Cx43. Purple arrow identifies cells expressing Cx43 and Cx30 but not GFAP. White arrows indicates cells expressing GFAP and Cx43 but not Cx30. Scale bar, 50 μm. Click here for file

Additional file 4 **Culture composition following mitogen or glial differentiating conditions.** Cultures were pulsed with BrdU 24 h prior to plating on a laminin substrate in the presence of EGF and FGF-2 (proliferative conditions) or RA and FBS (glial differentiation). Data are expressed as the percentage of cells actively proliferating at the time of plating that retained a Type1/2a NPC identity or specified to astrocytes, oligodendrocytes, or neurons. *p < 0.05, **p < 0.01, ANOVA, *post-hoc *Tukey test. Click here for file

Additional file 5 **Phosphorylation status of Cx43 in NPCs cultured as neurospheres.** Protein lysates were prepared from neurosphere cultures on DIV 14. Two predominant phosphoisoforms were detected corresponding to the P2 and P3 phosphovariants as well as hyperphosphorylated forms. Very low levels of the faster migrating unphosphorylated (NP) and little to no of the P1 phosphoisoform was detected. Data are representative of two independent cultures. Click here for file

Additional file 6 **Supplementary methodology describing genotyping, RT-PCR conditions, and antibodies employed.** Sequences, conditions, and dilutions. Click here for file

Additional file 7 **Schematic of experimental paradigm used to count cells in laminin-treated monolayer cultures.** Phase contrast (a) and GFAP immunoreactivity (b) of representative cultures plated under glial differentiating conditions on laminin. Five fields (shaded areas) per n = 5–10 cultures were obtained for quantification. Fields of interest were located 30 μm (white line) from the identified core of the neurosphere (black line). Click here for file
