# Supplementary figures and images for: The extracellular matrix controls gap junction protein expression and function in postnatal hippocampal neural progenitor cells
Source: BMC Neurosci. 2009 Feb 24;10:13. doi: 10.1186/1471-2202-10-13 (PMC2655299; doi:10.1186/1471-2202-10-13)

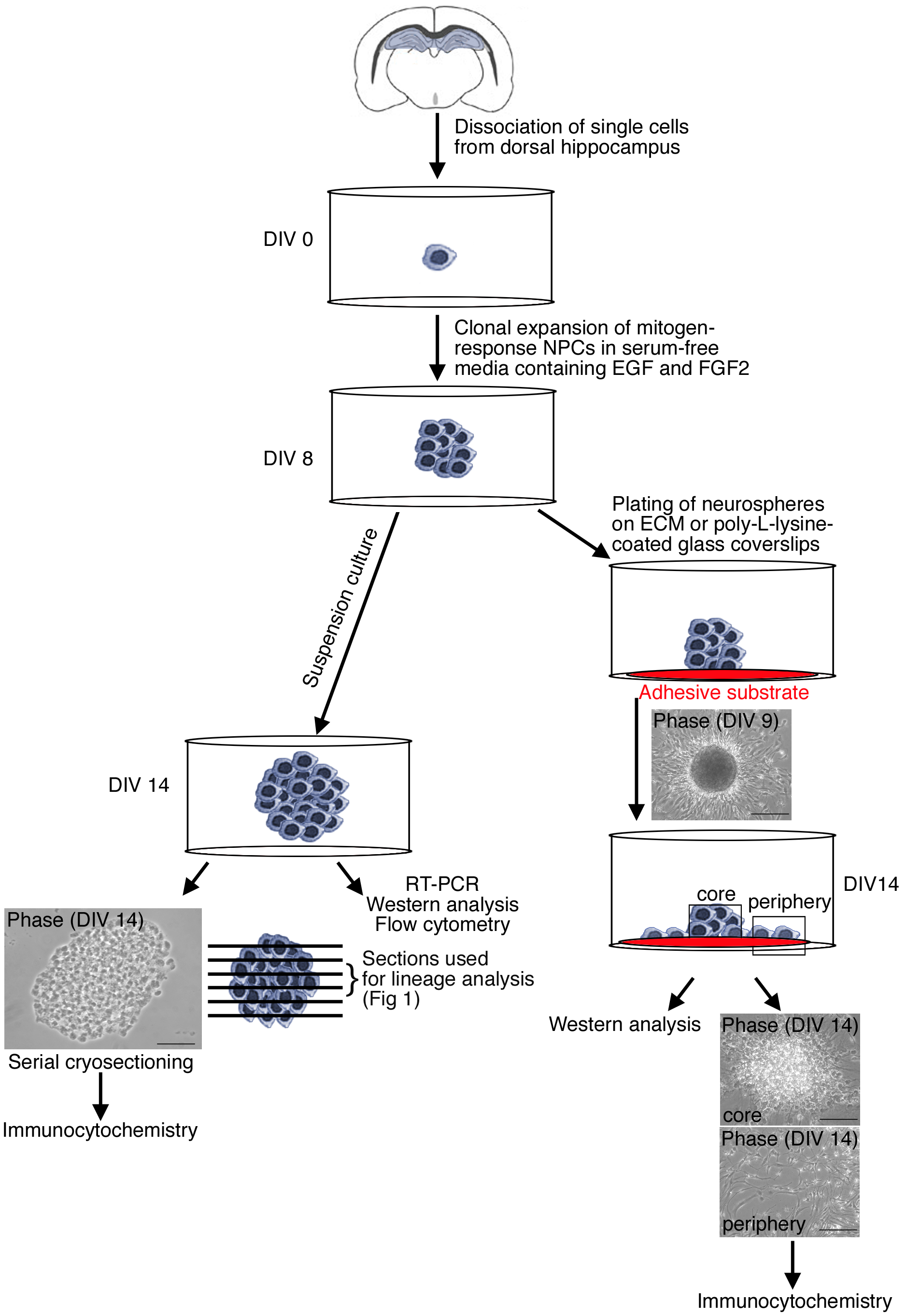

Supplement: Additional file 1 — Schematic representation of the neurosphere culture protocol and analysis. A graphic depiction of the protocol employed to culture and analyse neurospheres in the presence and absence of laminin. [file 1471-2202-10-13-S1.tiff]

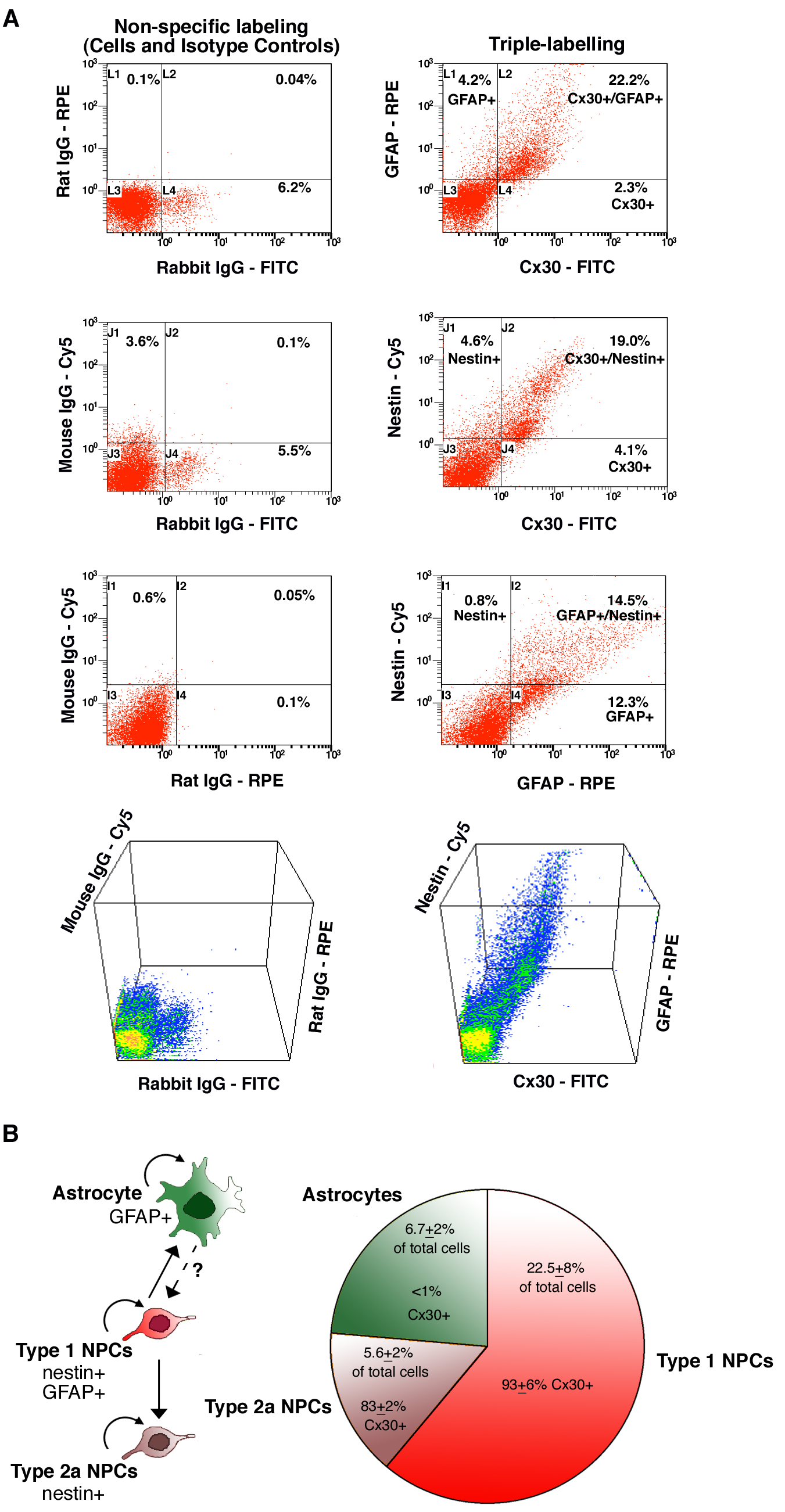

Supplement: Additional file 2 — Cx30 is expressed by Type 1 and 2a NPCs. (a) Hippocampal NPCs cultured as neurospheres were dissociated on DIV 14 and triple-labelled for Cx30, nestin, and GFAP. Two and three dimensional histograms from a representative flow cytometry analysis are presented for both isotype control (left panel) and experimental (right panel) reactions. The quadrant percentages represent the percentage of total cells expressing corresponding cell markers. (b) Quantitative analysis of Cx30 expression in Type 1 (GFAP+/nestin+) NPCs, Type 2a (GFAP-/nestin+) NPCs, and committed astrocytes (GFAP+/nestin-). The percentage of cells co-expressing Cx30 was calculated divided by the total percentage of each cell type by the total percentage of cells within each cell type expressing Cx30 in triple-labelling studies. Data are presented as mean ± SEM and represent the average of three independent flow cytometric analyses/cultures. [file 1471-2202-10-13-S2.tiff]

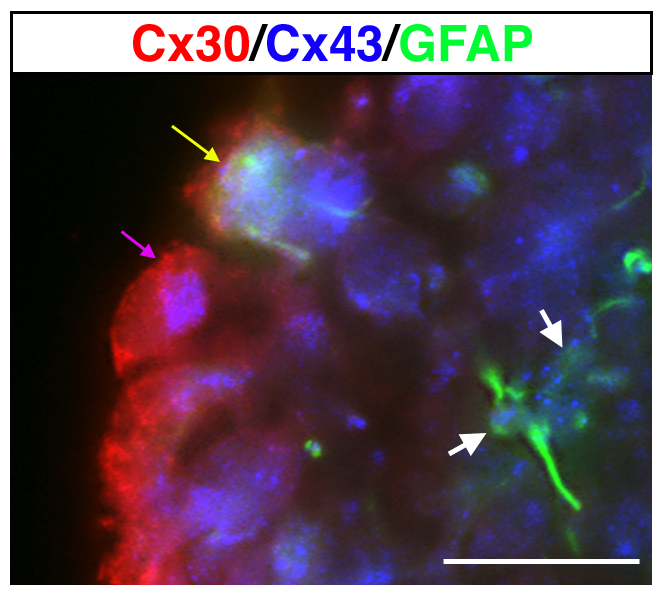

Supplement: Additional file 3 — Cx43 but not Cx30 is detected in all GFAP+ cell types. A 10 μm cyrosection through a neurosphere harvested on DIV 14 triple-labelled for Cx30, Cx43, and GFAP is presented. Yellow arrow points to cells expressing GFAP/Cx30/ and Cx43. Purple arrow identifies cells expressing Cx43 and Cx30 but not GFAP. White arrows indicates cells expressing GFAP and Cx43 but not Cx30. Scale bar, 50 μm. [file 1471-2202-10-13-S3.tiff]

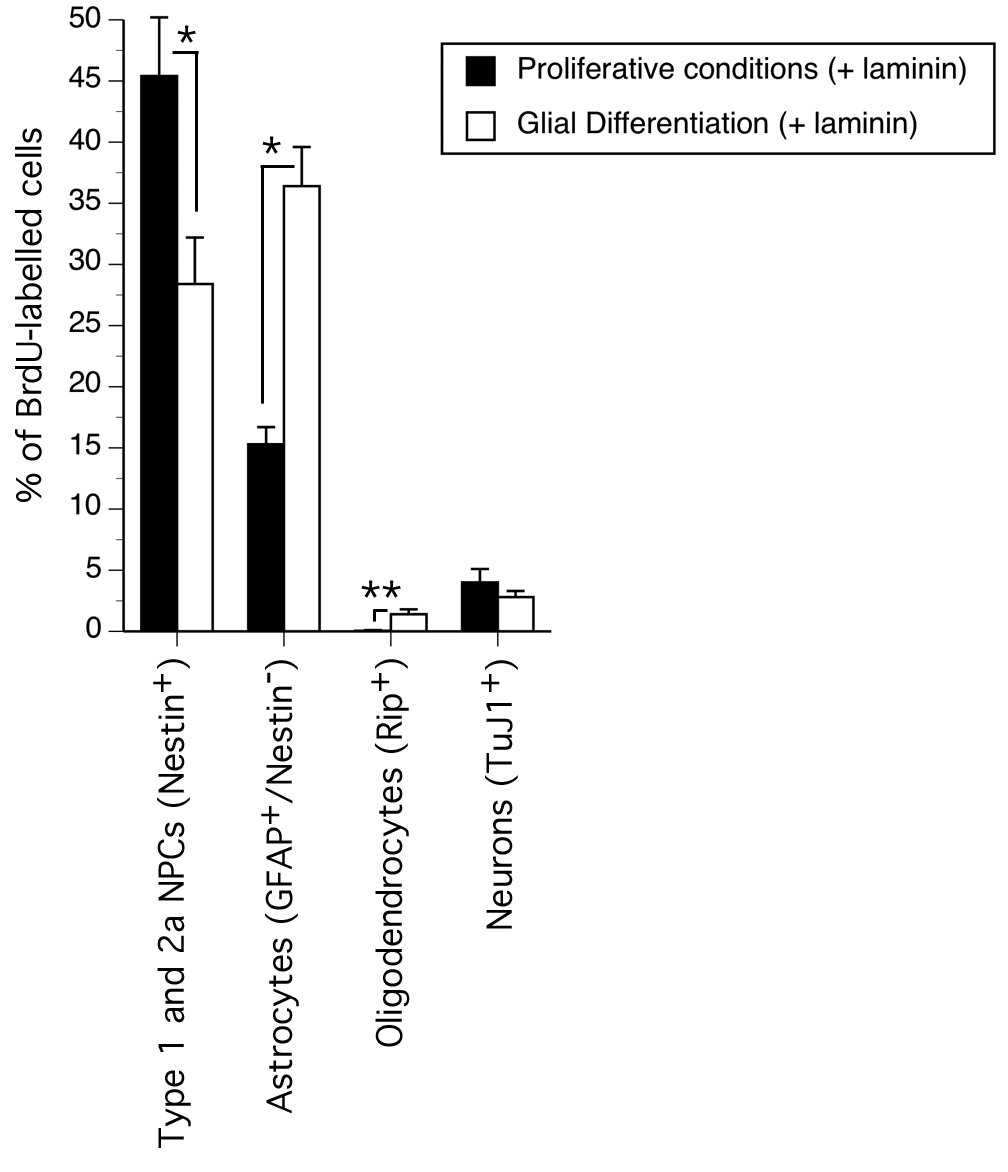

Supplement: Additional file 4 — Culture composition following mitogen or glial differentiating conditions. Cultures were pulsed with BrdU 24 h prior to plating on a laminin substrate in the presence of EGF and FGF-2 (proliferative conditions) or RA and FBS (glial differentiation). Data are expressed as the percentage of cells actively proliferating at the time of plating that retained a Type1/2a NPC identity or specified to astrocytes, oligodendrocytes, or neurons. *p < 0.05, **p < 0.01, ANOVA, post-hoc Tukey test. [file 1471-2202-10-13-S4.tiff]

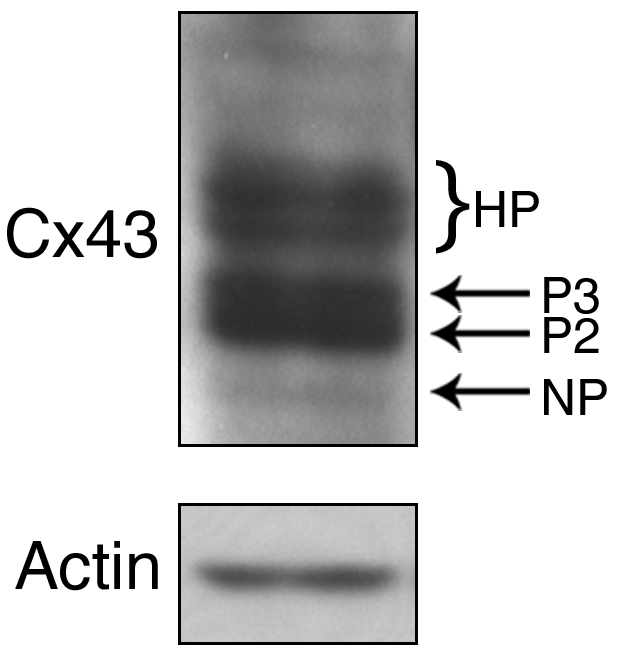

Supplement: Additional file 5 — Phosphorylation status of Cx43 in NPCs cultured as neurospheres. Protein lysates were prepared from neurosphere cultures on DIV 14. Two predominant phosphoisoforms were detected corresponding to the P2 and P3 phosphovariants as well as hyperphosphorylated forms. Very low levels of the faster migrating unphosphorylated (NP) and little to no of the P1 phosphoisoform was detected. Data are representative of two independent cultures. [file 1471-2202-10-13-S5.tiff]

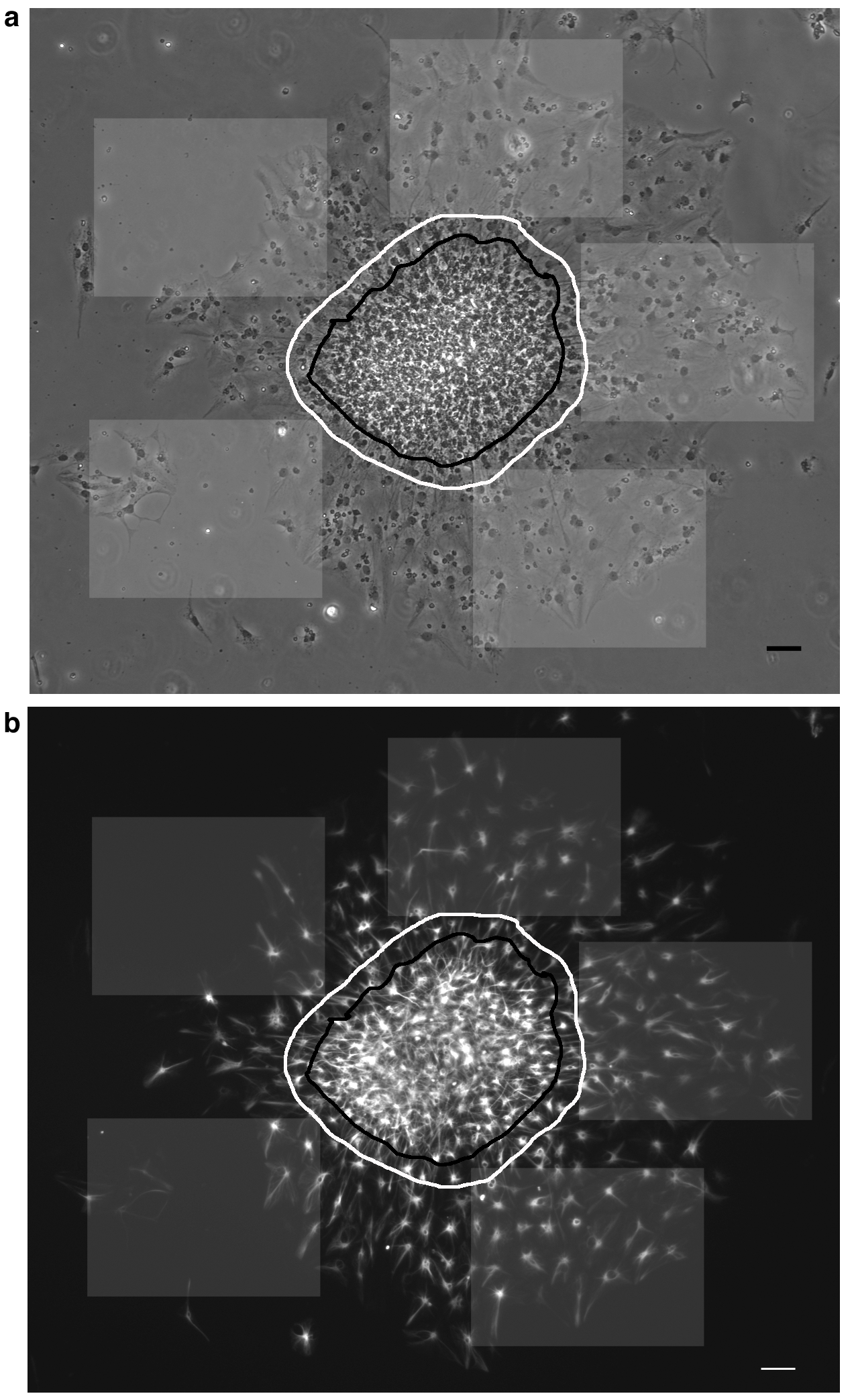

Supplement: Additional file 7 — Schematic of experimental paradigm used to count cells in laminin-treated monolayer cultures. Phase contrast (a) and GFAP immunoreactivity (b) of representative cultures plated under glial differentiating conditions on laminin. Five fields (shaded areas) per n = 5–10 cultures were obtained for quantification. Fields of interest were located 30 μm (white line) from the identified core of the neurosphere (black line). [file 1471-2202-10-13-S7.tiff]
