# Supplementary material for: The extracellular matrix controls gap junction protein expression and function in postnatal hippocampal neural progenitor cells
Source: BMC Neurosci. 2009 Feb 24;10:13. doi: 10.1186/1471-2202-10-13 (PMC2655299; doi:10.1186/1471-2202-10-13)
Supplement: Additional file 6 — Supplementary methodology describing genotyping, RT-PCR conditions, and antibodies employed. Sequences, conditions, and dilutions. [file 1471-2202-10-13-S6.doc]

**Supplementary Table 1. Genotyping protocols**

| **Gene** | **Reaction Conditions and Amplicon Sizes^a^** | **Cycling parameters** |
| --- | --- | --- |
| Cx29 | Cx29F: 5’-ATCTGTGCTGTGCTATTTGGAGT-3’  Cx29R: 5’-ACAGGTTGTGCTGCCAATAC-3’  lacZF: 5’-CCGACGGCACGCTGATTGAAG-3’  lacZR: 5’-ATGCGGTCGCGTTCGGTTGC-3’  Primer concentrations: 10 ng/µL each  Volume of tail DNA: 5 µL  Final reaction volume: 20 µL  (+ allele=700 bp)  (- allele=1100 bp) | 95^o^C 5 m  *30 cycles:*  94^o^C 15 s  66^o^C 15 s  72^o^C 80 s |
| Cx30 | F: 5’-GGTACCTCCTACTAATTAGCTTGG-3’  lacZR: 5’-AGCGAGTAACAACCCGTCGGATTC-3’  WTR: 5’-AGGTGGTACCCATTGTAGAGGAAG-3’  Primer concentrations: 8 ng/µL each  Volume of tail DNA: 1 µL  Final reaction volume: 25 µL  (+ allele=544 bp)  (- allele=460 bp) | 94^o^C 5 m  *35 cycles:*  92^o^C 45 s  60^o^C 45 s  72^o^C 45 s  72^o^C 10 m |
| Cx32 | F: 5’-ATACACCTTGCTCAGTGGCGTGAATCGGCA-3’  R: 5’-TCATTCTGCTTGTATTCAGGTGAGAGGCGG-3’  Primer concentrations: 8 ng/µL each  Volume of tail DNA: 2.5 µL  Final reaction volume: 12.5 µL  (+ allele=750 bp) | 95^o^C 10 m  *30 cycles:*  95^o^C 60 s  67^o^C 60 s  72^o^C 60 s |
|  | F: 5’-TCTTACTCCACACAGGCATAGAGTGTCTGC-3’  R: 5’-TCATTCTGCTTGTATTCAGGTGAGAGGCGG-3’  Primer concentrations: 8 ng/µL each  Volume of tail DNA: 2.5 µL  Final reaction volume: 12.5 µL  (- allele=1300 bp) | 95^o^C 10 m  *30 cycles:*  95^o^C 60 s  67^o^C 60 s  72^o^C 60 s |
| Cx36 | F: 5’-AGCGGAGGGAGCAAACGAGAAG-3’  R: 5’-CTGCCGAAATTGGGAACACTGAC-3’  Primer concentrations: 8 ng/µL each  Volume of tail DNA: 5 µL  Final reaction volume: 25 µL  (+ allele=533 bp) | 94°C 60s  *30 cycles:*  94°C 15 s  69°C 15 s  72°C 45 s |
|  | PLAPF: 5’-GGTGAACCGCAACTGGTACT-3’  PLAPR: 5’-CCCACCTTGGCTGTAGTCAT-3’  Primer concentrations: 0.5 µM each  Volume of tail DNA: 5 µL  Final reaction volume: 20 µL  (- allele=187 bp) | 95^o^C 15m  *35 cycles:*  95^o^C 30s  63^o^C 90s  72^o^C 2m  72^o^C 15m |
| Cx37^b^ | F: 5’-TGCTAGACCAGGTCCAGGAAC-3’  neoR: 5’-AGAGGCTATTCGGCTATGACT-3’  WTR: 5’-GTCCCTTCGTGCCTTTATCTC-3’  Primer concentrations: 125 nM each  Volume of tail DNA: 1.5 µL  Final reaction volume: 20 µL  (+ allele=750 bp)  (- allele=1300 bp) | 94^o^C 3 m  *30 cycles:*  94^o^C 30 s  63^o^C 30 s  72^o^C 90 s  72^o^C 5 m |
| Cx40^c^ | F: 5’-TGGAGCCACAGTTGCAATGGT-3’  neoR: 5’-GCACGAGACTAGTGAGACGTG-3’  WTR: 5’-TCTCTGACTCCGAAAGGCAAG-3’  Primer concentrations: 20 ng/µL each  Volume of tail DNA: 2 µL  Final reaction volume: 30 µL  (+ allele=270 bp)  (- allele=470 bp) | 94^o^C 3 m  *30 cycles:*  94^o^C 30 s  64^o^C 30 s  72^o^C 30 s  72^o^C 4 m |
| Cx45^d^ | CreTK139F: 5’-ATTTGCCTGCATTACCGGTC-3’  CreTK141R: 5’-ATCAACGTTTTGTTTTCGGA-3’  Primer concentrations: 4 ng/µL each  Volume of tail DNA: 2.5 µL  Final reaction volume: 12.5 µL  (Cre transgene=300 bp) | 94^o^C 5m  *30 cycles:*  94^o^C 30 s  56^o^C 30 s  72^o^C 60 s |
|  | Cx45F: 5’-CTTGGCTTCCTTAATTACTTTA-3’  Cx45R: 5’-CTTCCCTACAAATGTCGAATG-3’  neoR: 5’-AGGGGACGAAGACAGTAT3’  Primer concentrations: 0.8 µg/µL each  Volume of tail DNA: 2.5 µL  Final reaction volume: 12.5 µL  (+ allele=570 bp)  (Flx allele=610 bp  (- allele=820 bp) | 94^o^C 5m  *30 cycles:*  94^o^C 30 s  56^o^C 30 s  72^o^C 60 s |
| Cx47^e^ | F: 5’-AAGGCTGGTGCTGCTGGAAT-3’  R: 5’-TGACCACCGTCTTGCCATCA-3’  Primer concentrations: 0.4 µM each  Volume of tail DNA: 5 µL  Final reaction volume: 25 µL  (+ allele=1100 bp) | 95^o^C 5 m  *35 cycles:*  95^o^C 15 s  67^o^C 15 s  72^o^C 3 m |
|  | F: 5’-TCGCATTGTCTGAGTAGGTGTC-3’  R: 5’-CAGAGTTCCTCTGCACAGAGAT-3’  Primer concentrations: 10 ng/µL each  Volume of tail DNA: 5 µL  Final reaction volume: 20 µL  (- allele=2200 bp) | 95^o^C 15 m  *35 cycles:*  95^o^C 30 s  64^o^C 90 s  72^o^C 2 m  72^o^C 15m |

^a^Unless otherwise stated, PCR reactions contain 1X Advantage 2 PCR buffer, 0.8 mM dNTPs and 0.4 µL of Advantage 2 Taq polymerase (Clontech)

^b^Final concentration of dNTPs is 0.2 mM.

^c^Final concentration of dNTPs is 0.2 mM. Reaction uses Titanium Taq Polymerase in 1X Titanium PCR Buffer

^d^Reactions use Titanium Taq Polymerase in 1X Titanium PCR Buffer.

## ^e^For the + allele, final Advantage 2 PCR buffer concentration is 0.8X.

**Supplementary Table 2. RT-PCR protocols^a^**

| **Gene** | **Primer Sequence** | **Primer Concentration** | **Amplicon length** |
| --- | --- | --- | --- |
| Cx26 (GJB2) | F: 5’-GGATGTGGCAGTCAGTATCA  R: 5’-TCTTGGCAGGAAGAAGTGTC | 0.5 µM | 368 bp |
| Cx29 (GJC3) | F: 5’-GGTTTTCGGCAATGAT  R: 5’-AGAAGCTTGAGGCTTTTAGC | 4 ng/µL | 278 bp |
| Cx30 (GJB6) | F: 5’-GCCAGGGTGCAAGAACGTCTGC  R: 5’-GGCATGGTTGGGTGGTTTCTC | 10 ng/µL | 535 bp |
| Cx32 (GJB1)^b^ | F: 5’-GTGGCGTGAATCGGCACTCTAC  R: 5’-CTCCGCCACGTTGAGGATAATG | 10 ng/µL | 593 bp |
| Cx36 (GJD2) | F: 5’-AGCGGAGGGAGCAAACGAGAAG  R: 5’-CTGCCGAAATTGGGAACACTGAC | 10 ng/µL | 533 bp |
| Cx37 (GJA4) | F: 5’-AGAGCGGTTGCGGCAGAAAGAGG  R: 5’-TGGATGAGAGCCCGTTGTAGGTG | 10 ng/µL | 551 bp |
| Cx40 (GJA5) | F: 5’-TTTGGCCAAGTCACGGCAGGG  R: 5’-TTGTCACTGTGGTAGCCCTGAGG | 4 ng/µL | 311 bp |
| Cx43 (GJA1) | F: 5’-CCTGCCGCAATTACAACAAG  R: 5’-AAGGTCGCTGATCCACGATA | 10 ng/µL | 201 bp |
| Cx45 (GJC1)^b^ | F: 5’-GAGGTGGGCTTTCTAATAGGGCAG  R: 5’-ATGGGGGTTGTTTTGGTGATGG | 10 ng/µL | 528 bp |
| Cx47 (GJC2) | F: 5’-GCTGGAGGAGATCCACAATCATTC  R: 5’-GTGTGGAGATGACCACTATCTGGA | 10 ng/µL | 233 bp |
| GAPDH^b^ | F: 5’-TGGTGCTGAGTATGTCGTGGAGT  R: 5’-AGTCTTCTGAGTGGCAGTGATGG | 0.2 µM | 292 bp |

^a^All reactions contained 1 µL of RT product, 0.8 mM dNTPs, 1X PCR Buffer, 1X Advantage 2 Taq Polymerase in a reaction volume of 25 µL except Cx32 and GAPDH reactions. Cycling parameter: 94^o^C 5 m, 35 cycles of 94^o^C 25 s, 59^o^C 50 s, 72^o^C 1 m 45 s and a final extension of 72^o^C 7 m.

^b^1X Titanium Taq Polymerase was used.

## Supplementary Table 3. List of primary and secondary antibodies used for immunocytochemistry and Western Blotting

| Antibody | Type | Species | Source | Dilution  Immuno | Dilution  Western | Dilution  Flow |
| --- | --- | --- | --- | --- | --- | --- |
| Cx26 | Polyclonal | Rabbit | Zymed | 1:25 | 1:100 | --- |
| Cx29 | Polyclonal | Rabbit | Dr.David Paul | 1:20 | --- | --- |
| Cx30 | Monoclonal | Mouse | Zymed | 1:50 | --- | 1:25 |
| Cx30 | Polyclonal | Rabbit | Zymed | --- | --- | 1:25 |
| Cx32 | Monoclonal | Mouse | Zymed | 1 µg/mL | 1:250 | --- |
| Cx36 | Polyclonal | Rabbit | Zymed | 5 µg/mL | 1:100 | --- |
| Cx37 | Polyclonal | Rabbit | Dr. Alex Simon | 1:200 | --- | --- |
| Cx40 | Polyclonal | Rabbit | Zymed | 1:50 | --- | --- |
| Cx43 | Monoclonal | Mouse | Chemicon | 1:100 | --- | 1:1000 |
| Cx43 | Polyclonal | Rabbit | Zymed | 1:100 | --- | --- |
| Cx45 | Polyclonal | Rabbit | Chemicon | 1:1000 | --- | --- |
| Cx47 | Polyclonal | Rabbit | Dr. David Paul | 1:250 | --- | --- |
| Actin | Monoclonal | Mouse | Sigma | --- | 1:1000 | --- |
| β-galactosidase | Polyclonal | Rabbit | Chemicon | 1:1500 | --- | --- |
| BrdU | Monoclonal | Mouse | Roche | 6 µg/mL | --- | --- |
| DCX | Polyclonal | Guinea Pig | Chemicon | 1:4000 | --- | --- |
| GFAP | Polyclonal | Rabbit | Sigma | 1:100 | --- | --- |
| GFAP | Monoclonal  Cy-3 conjugated | Mouse | Sigma | 1:800 | --- | --- |
| GFAP | Monoclonal | Rat | Zymed | 1:40 | --- | 1:25 |
| Nestin | Monoclonal | Mouse | Chemicon | 1:50 | --- | 1:50 |
| NCAM | Monoclonal | Mouse | Sigma | 1:400 | --- | --- |
| NG2 | Polyclonal | Rabbit | Chemicon | 1:200 | --- | --- |
| NeuN | Monoclonal | Mouse | Chemicon | 1:100 | --- | --- |
| PDGFαR | Monoclonal | Rat | BD | 1:300 |  |  |
| RIP | Monoclonal | Mouse | Chemicon | 1:1000 | --- | --- |
| TuJ1 (βIII-tubulin) | Monoclonal | Mouse | Research Diagnostics | 1:250 | --- | --- |
| Mouse IgG | Cy3-conjugated | Donkey | Jackson | 1:800 | --- | --- |
| Rabbit IgG | Cy3-conjugated | Donkey | Jackson | 1:600 | --- | --- |
| Mouse IgG | FITC-conjugated | Donkey | Jackson | 1:100 | --- | --- |
| Rabbit IgG | FITC-conjugated | Donkey | Jackson | 1:100 | --- | 1:50 |
| Rat IgG | FITC-conjugated | Donkey | Jackson | 1:80 |  |  |
| Mouse IgG | HRP-conjugated | Donkey | Jackson | --- | 1:2000 | --- |
| Rabbit IgG | HRP-conjugated | Donkey | Jackson | --- | 1:5000 | --- |
| Mouse IgG | Cy5-  conjugated | Goat | Invitrogen | --- | --- | 1:50 |
| Rat IgG | RPE-  conjugated | Goat | Serotec | --- | --- | 1:10 |
| Rabbit IgG | AMCA-  conjugated | Goat | Jackson | 1:100 | --- | --- |
| Mouse IgG | AMCA-  conjugated | Donkey | Jackson | 1:100 | --- | --- |
